# History of a Case of Tinea Capitis Cured by Vaccination

**Published:** 1807-10

**Authors:** 

**Affiliations:** Philadelphia


### *History of a Case of Tinea Capitis cured by Vaccination; by* Dr. Coxe, *of Philadelphia.*

Eliza Moore, a mulatto child of two years of age, was brought to me March 28, 1804, for advice in an obstinate case of tinea capitis, which had succeeded a sudden suppression of a discharge from the ears, and had now continued nearly seven months. Little or nothing had been done for the child, and the disease had extended itself over the whole head, which had acquired an unpleasant appearance and smell, from the matting together of the hair by the discharge, and want of due attention to it in point of cleanliness. As the small-pox existed in the neighbourhood, and in the very house where the child resided, I told the mother it would be proper first to vaccinate her, to secure her from that disease, before we attended to the complaint of the head. I had another object besides this in view, which was, an expectation of the tinea being removed by the operation of the vaccine on her system. I therefore vaccinated her by two punctures, and directed the mother merely to wash the head with soap and water. This attempt failed, and also a second on the 2d of April; a third, on the 5th, proved more successful, and the disease advanced with perfect regularity, though slightly retarded by early rubbing. It soon, however, resumed its genuine appearance, and the areola was perceptible about the tenth, or beginning of the eleventh day, day, attended by a slight degree of fever. At this period a very evident alteration in the disease of the head took place. It began to dry up, and in a few days the discharge from the ears recurred; by the time the scab was perfectly complete, about the fifteenth or sixteenth day, the disease was nearly gone, the incrustations having come away in large pieces, leaving the head perfectly sound beneath. The running of the ears in a few weeks was suspended, without any recurrence of the tinea; and the child continues perfectly well to the present time. After the ninth or tenth day, I desired the mother to expose her as much as possible to the small-pox. This she did without any effect from it.

